# Supplementary material for: The Magee 3 Equation Predicts Favorable Pathologic Response to Neoadjuvant Endocrine Therapy in Breast Cancer Patients
Source: Cancers (Basel). 2024 Jan 13;16(2):339. doi: 10.3390/cancers16020339 (PMC10813970; doi:10.3390/cancers16020339)
Supplement: Supplementary file 1 [file cancers-16-00339-s001.zip › cancers-2650255-supplementary.pdf]

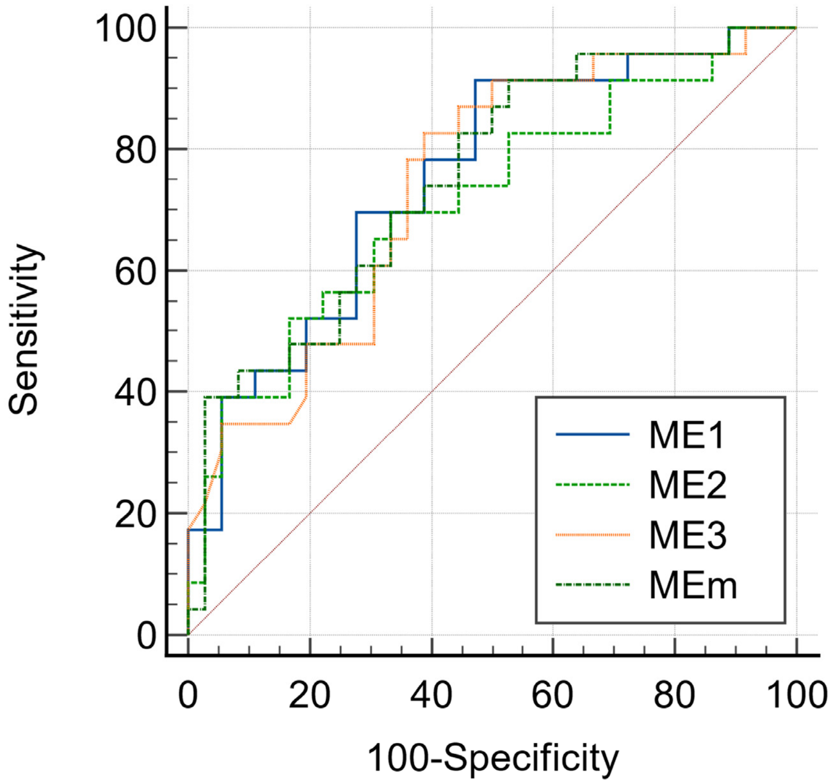

Figure S1: Comparison between ROC curves of ME1, ME2, ME3, and MEm scores in a sample of breast cancer patients undergoing neoadjuvant endocrine therapy.

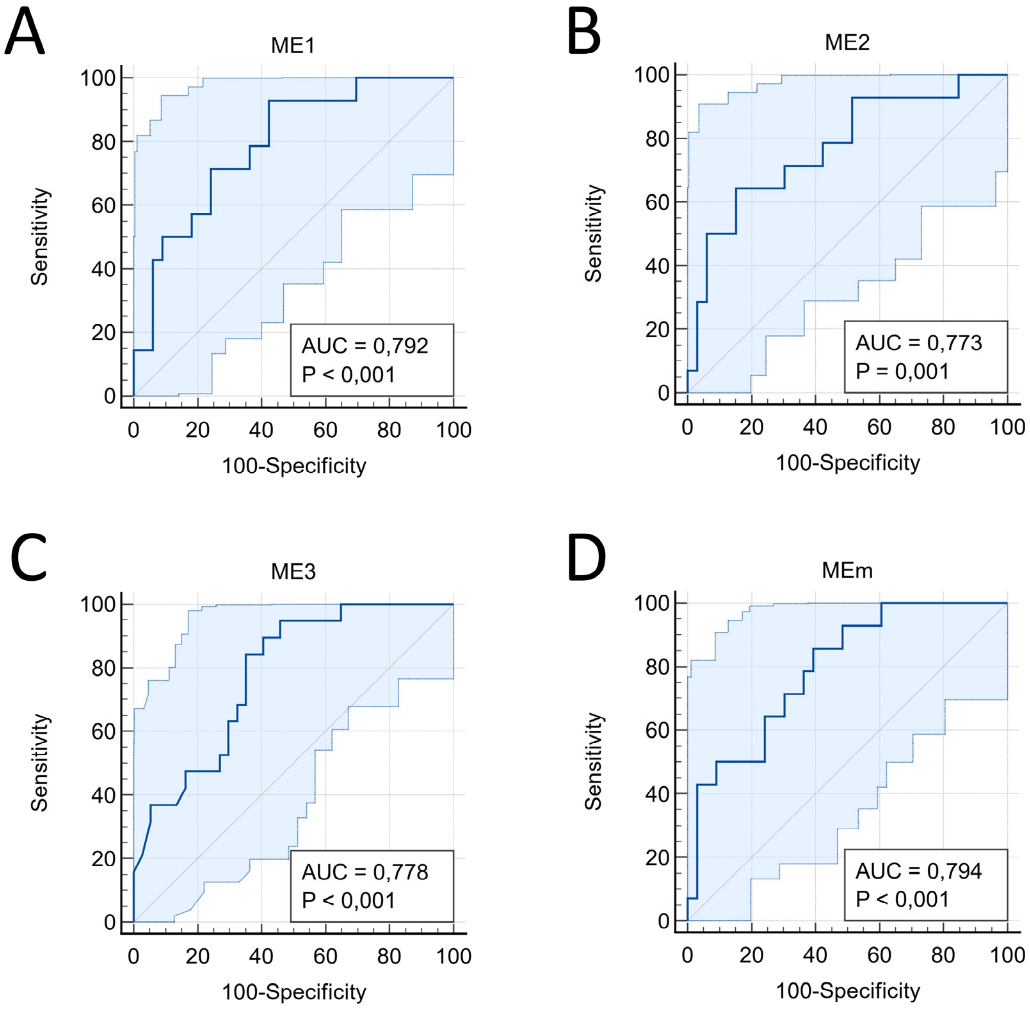

Figure S2: ROC curves with their respective confidence intervals and AUC values in the sample of breast cancer patients, excluding those with clinical stage IA tumors. (A) ME1; (B) ME2; (C) ME3; (D) MEm.

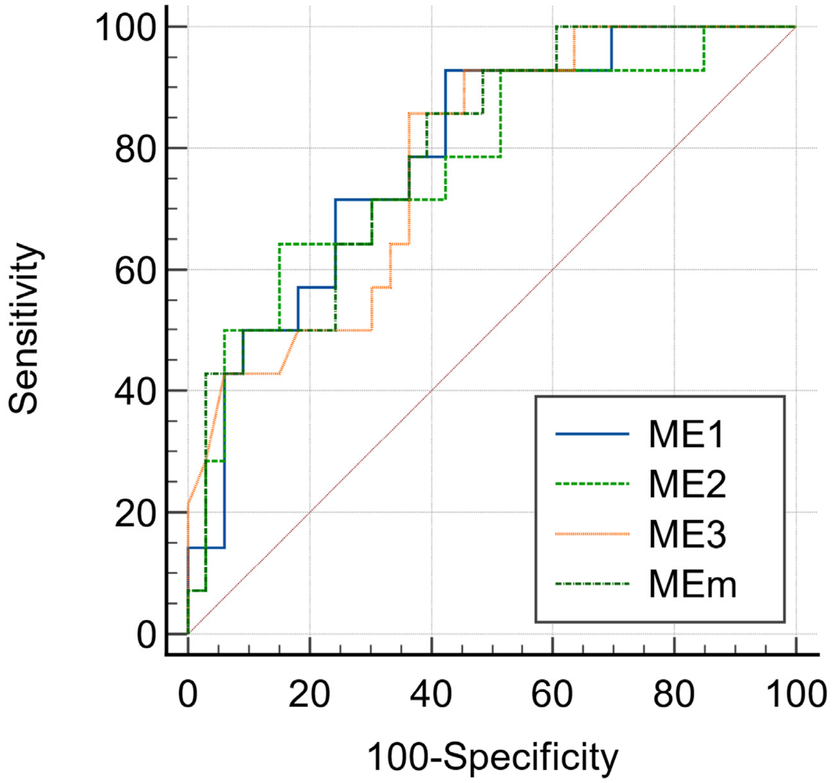

Figure S3: Comparison between ROC curves of ME1, ME2, ME3, and MEm scores in the sample of breast cancer patients undergoing neoadjuvant endocrine therapy (excluding those with clinical stage IA tumors).
